# ﻿*Thismia selangorensis* (Thismiaceae): a new mitriform fairy lantern species from Selangor, Malaysia

**DOI:** 10.3897/phytokeys.267.157968

**Published:** 2025-11-28

**Authors:** Mat Yunoh Siti-Munirah, Tan Gim Siew, Mohd Faizal Mat-Tahir, Ahmad Azhar

**Affiliations:** 1 Forest Research Institute Malaysia, 52109 Kepong, Selangor, Malaysia Forest Research Institute Malaysia Kepong Malaysia; 2 Taman Tropicana Cheras, 43000 Kajang, Selangor, Malaysia Taman Tropicana Cheras Kajang Malaysia; 3 Universiti Kebangsaan Malaysia, 43500 Bangi, Selangor, Malaysia Universiti Kebangsaan Malaysia Bangi Malaysia; 4 Selangor State Forestry Department, 40660 Shah Alam, Selangor, Malaysia Selangor State Forestry Department Shah Alam Malaysia

**Keywords:** Ecopark, endemic, *
Geomitra
*, mitre, achlorophyllous plant, Southeast Asian flora, taxonomy

## Abstract

*Thismia selangorensis*, a distinct mitriform species of the mycoheterotrophic genus *Thismia*, is described herein. It was first discovered in a tree hole on a riverbank in Taman Eko Rimba Sungai Chongkak, Selangor, Malaysia. This new species is superficially similar to members of Thismia section Geomitra in that it has coralliform roots, inner tepals forming a mitre with three appendages on top, and stamens with a prominent dorsal rib. However, *T. selangorensis* differs from known species of T. sect. Geomitra in several morphological features, including the colour of the flowers, the shape of the mitre, the shape of the inner tepal lobes forming the mitre, and the presence of translucent reticulation on the inner surface of the floral tube. *Thismia selangorensis* is provisionally classified as Critically Endangered according to the IUCN Red List categories and criteria.

## ﻿Introduction

The family Thismiaceae currently consists of five genera – *Haplothismia* Airy Shaw, *Oxygyne* Schltr., *Thismia* Griff., *Tiputinia* P.E. Berry & C.L. Woodw., and *Relictithismia* Suetsugu & Tagane (Cheek et al. 2023; Suetsugu et al. 2024) – with a total of 127 species (Cheek et al. 2023; Suetsugu et al. 2024; Dančák et al. 2025; POWO 2025; Siti-Munirah and Mohamad Alias 2025). Of these, *Thismia* is the most diverse, with 120 species (Dančák et al. 2025; POWO 2025; Siti-Munirah and Mohamad Alias 2025), making it the largest genus of mycoheterotrophic plants known to science. *Thismia* is a genus of non-photosynthetic, mycoheterotrophic, and monocotyledonous herbs. These plants do not have chlorophyll and are parasites entirely dependent on exploiting mycorrhizal mutualism (Leake 1994; Merckx 2013; Ogura-Tsujita et al. 2021; Suetsugu et al. 2022). *Thismia* species are usually found in undisturbed, shady forest soils, often in leaf-litter-rich, moist microhabitats of tropical lowland to montane forests, where their tiny, ephemeral flowers appear unpredictably (Kiew 1999; Kumar et al. 2017; Engels et al. 2022; Siti-Munirah and Dome 2022). The unpredictable occurrence of mycoheterotrophs is due to their predominantly subterranean lifestyle (Leake 1994). Even if a population is recorded at a site, its discovery on subsequent visits is not guaranteed, as individuals may remain hidden unless they are in the flowering or fruiting phase, making them extremely difficult to find (Merckx et al. 2013; Dančák et al. 2020a). The number of recognised species within *Thismia* has increased considerably in recent years (Dančák et al. 2020b).

Prior to this study, *Thismia* was represented by only two species in Selangor, i.e. *Thismia alba* Holttum & Jonker and *T. fumida* Ridl. *Thismia alba* was first discovered in Pahang and later recorded throughout Peninsular Malaysia (e.g. in Pahang, Terengganu, and Kelantan) (Jonker 1948; Siti-Munirah and Mohamad Alias 2025). In recent years, this species has only been sighted a few times in forested areas of the Forest Research Institute of Malaysia (FRIM) in Selangor. *Thismia fumida* was described by Henry Nicholas Ridley in 1890 and was also later recorded in Singapore (Ridley 1890; Stone 1980). However, the type locality is given as Petaling (Selangor), which is now mainly an urban area. No new sightings have been reported to date. Additionally, three other unknown *Thismia* species that are new to science have been found in Selangor in recent years. One is described here, and the other two, which do not belong to the *Geomitra* group, will be described in a separate publication.

In November 2023, during a routine photographic activity in Taman Eko Rimba (TER) Sungai Chongkak – part of the Hulu Langat Forest Reserve (FR) in Selangor – a naturalist, Gim Siew (second author), discovered an unidentified *Thismia* species growing hidden near the desk roots (buttress) of a tree on the bank of the Sungai Chongkak. Although the area has been a well-known and frequently visited picnic and recreation site for decades, no such species has been documented to date. Following its discovery, we jointly conducted a field study to collect specimens for further botanical research, herbarium preservation, and taxonomic analysis. A morphological examination of the floral tube and tepals revealed a unique combination of features that did not match any previously described *Thismia* species, leading to the official description of *Thismia selangorensis* Siti-Munirah & Gim Siew.

## ﻿Materials and methods

**Figure 1.**
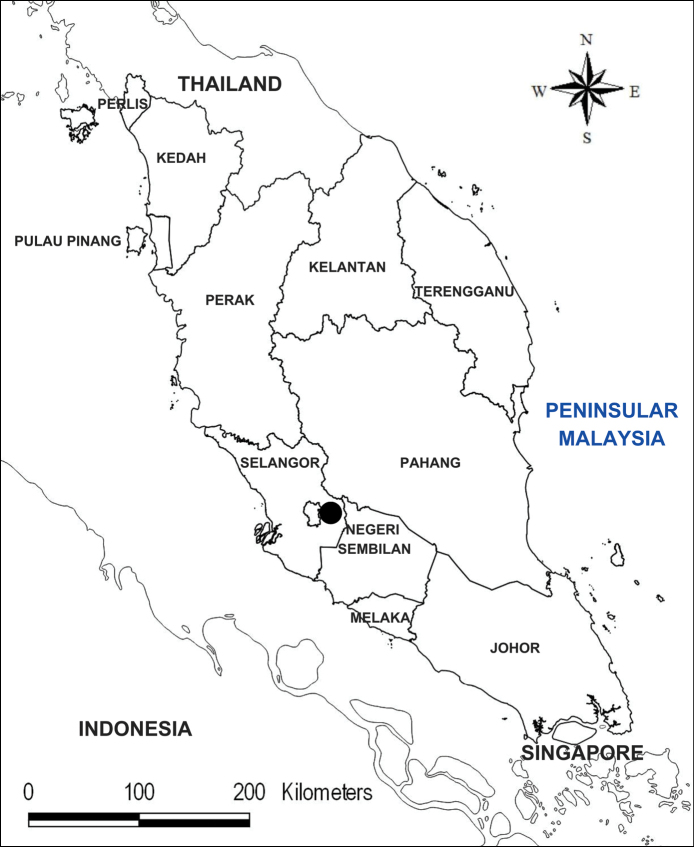
Map of the Malay Peninsula showing the location of TER Sungai Chongkak (dot), the type locality of *Thismia selangorensis*.

This study is based on material collected in TER Sungai Chongkak, Hulu Langat FR, Hulu Langat District, Selangor, Malaysia (Fig. 1). Morphological features were studied using stereomicroscopy and high-resolution macrophotography. Measurements were made using fresh and liquid-preserved material. The specimen was thoroughly compared with original drawings and descriptions in the protologues of Thismia sect. Geomitra (Chantanaorrapint and Chantanaorrapint 2009; Tsukaya and Okada 2012; Siti-Munirah 2018; Suetsugu et al. 2018; Chantanaorrapint and Seelanan 2021; Siti-Munirah et al. 2022).

## ﻿Taxonomic account

### ﻿ Thismia selangorensis

Taxon classificationPlantaeDioscorealesBurmanniaceae

Siti-Munirah & Gim Siew sp. nov.

91A42607-3636-5FF8-A19A-AE5F4985616E

urn:lsid:ipni.org:names:77372570-1

Figs 2, 3, 4

#### Diagnosis.

*Thismia selangorensis* differs from other species of Thismia sect. Geomitra in the following combinations of characteristics: the flower is white to brownish with peach colouration; the distal part of inner tepals is arrow-like with distinct basal lobes, perfectly connate to each other when young, partly splitting on the sutures with age; mitre is wide and resembling a large umbrella, convex and trilobed when viewed from above in younger flowers, flat and irregularly hexalobed in old flowers.

**Figure 2.**
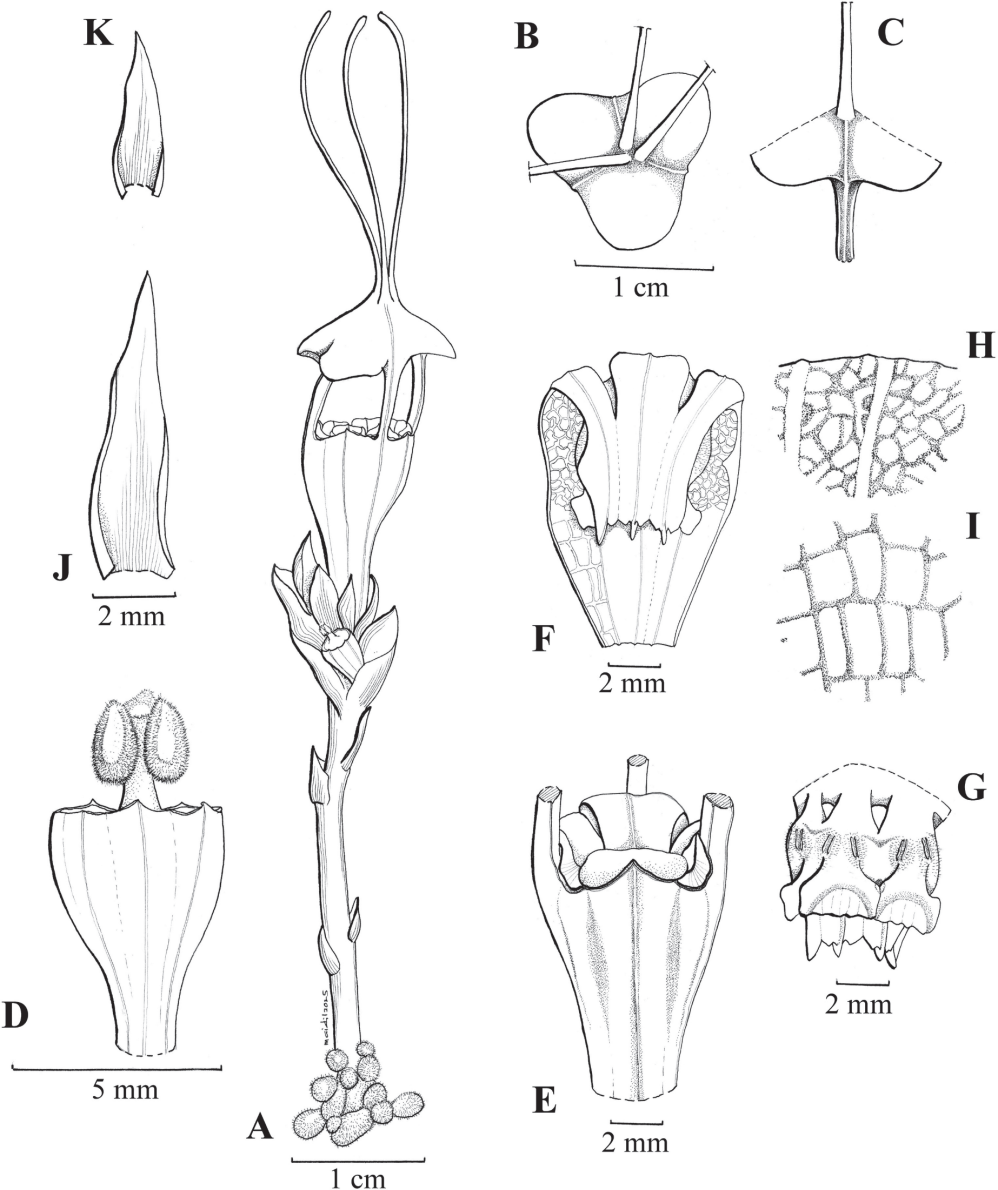
*Thismia selangorensis*. **A.** Plants with flowers and roots; **B.** Top view of mitre (inner tepals); **C.** Side view of inner tepal; **D.** Ovary, stigma, and style; **E.** Floral tube with mitre removed and minute triangular outer tepal; **F.** Longitudinal section of floral tube with three stamens; **G.** Outer view of three pendulous stamens; **H, I.** View of inner side of floral tube, upper part (**H**), lower part (**I**); **J.** Bract; **K.** Leaf. Drawn by Mohammad Aidil Nordin from the spirit material (FRI 79179 & 79180).

#### Type.

Malaysia. Peninsular Malaysia: Selangor, Hulu Langat District, Taman Eko Rimba Sungai Chongkak, ca. 184 m elev., 27 November 2023, Siti-Munirah MY, Gim Siew T & Mohd Faizal MT, FRI 79179 (holotype KEP!, spirit collection, barcode no. SC13204).

Achlorophyllous ground herb, ca. 10 cm tall. ***Roots*** short, clustered, coralliform, light brown to whitish, surface covered with trichomes. ***Stem*** ca. 3 cm tall, ca. 2–3 mm in diameter, erect, unbranched, light brown, glabrous. ***Leaves*** scale-like, spirally arranged, denser at the stem tip, up to 4 mm long and 1–2 mm wide at the base, triangular-ovate to lanceolate, with pointed to acuminate tip, pale brown, glabrous. ***Bracts*** crowded, scale-like, ca. 1 cm long, 2 mm wide at the base, slightly larger than the upper leaves; triangular-ovate to lanceolate, apex acute to acuminate, brown. ***Pedicels*** ca. 2 cm long, 2–3 mm wide during the anthesis. ***Inflorescence*** near the top of stem. ***Flower*** solitary or in clusters, up to 6 cm long (including the ovary, floral tube and appendages). ***Perianth*** actinomorphic, with 6 tepals fused into a floral tube that develops into an umbrella-shaped mitre with 3 slender, claviform appendages at the tip. ***Floral tube*** narrowly urceolate, ca. 16 mm long and 4–6 mm wide, constricted above the ovary and widest in the upper part; ***outer surface*** white, peach to brownish, with 12 pale brown longitudinal stripes on the upper part, with 6 longitudinal ribs, glabrous; ***inner surface*** with 12 brown longitudinal ribs, without transverse bar but covered by a translucent white reticulation. ***Tepals*** 6; ***outer tepals*** 3, erect, minute, triangular, brownish; ***inner tepals*** 3, erect, narrowly stemmed and broadly lobed (arrow-like), apically connate and forming a mitre shape together; ***mitre*** dome to umbrella-like, convex in younger flowers and flatten as they mature (with increasing age the lobes split and change shape during anthesis), white-brownish-peach, glabrous, somewhat papery, with 3 lateral, rectangular openings, each up to ca. 10 mm tall, ca. 6 mm wide; ***mitre appendages*** 3, up to 3 cm long, 1 mm wide, broad and flattened at the base and forming a furrow in the middle of the mitre, with a club-shaped tip, brown, paler toward base and tip, glabrous. ***Annulus*** absent. ***Stamens*** 6, hanging down from the apical edge of the floral tube; ***filaments*** dark-grey-whitish to bluish coloured, bent downwards, with the bases protruding slightly beyond the apex of the floral tube, not fused, forming 6 openings visible from above. ***Connectives*** broad, bluish, fused laterally into a tube, ca. 8 mm long, each connective with conspicuous longitudinal rib extending along the entire length of the inner surface (convex on the inner surface, concave on the outer surface); supraconnective apex with a longer central lobe (an extension of the longitudinal rib) and two smaller lateral lobes with pointed tips, all glabrous; ***lateral appendage*** box-shaped, bright blue, projecting towards the floral tube without reaching the supraconnective apex, glabrous on the surface and sparsely hairy at the horn-like corners; ***thecae*** 2 on each stamen, elongated, ca. 0.8 mm long, blue-whitish; ***interstaminal glands*** elliptic-oblong, translucent, inserted at the fusion line between the connective tissues. ***Ovary*** obconical, ca. 5 mm long, 4 mm wide, dark brown, with 6 longitudinal ribs; ***placentae*** 3; ***style*** short, ca. 1 mm long, blackish; ***stigma*** 3-lobed, blackish, each lobe oblong, ca. 1 mm long and slightly bifid at the apex, the surface slightly papillose. ***Fruit*** a cup-shaped capsule.

**Figure 3.**
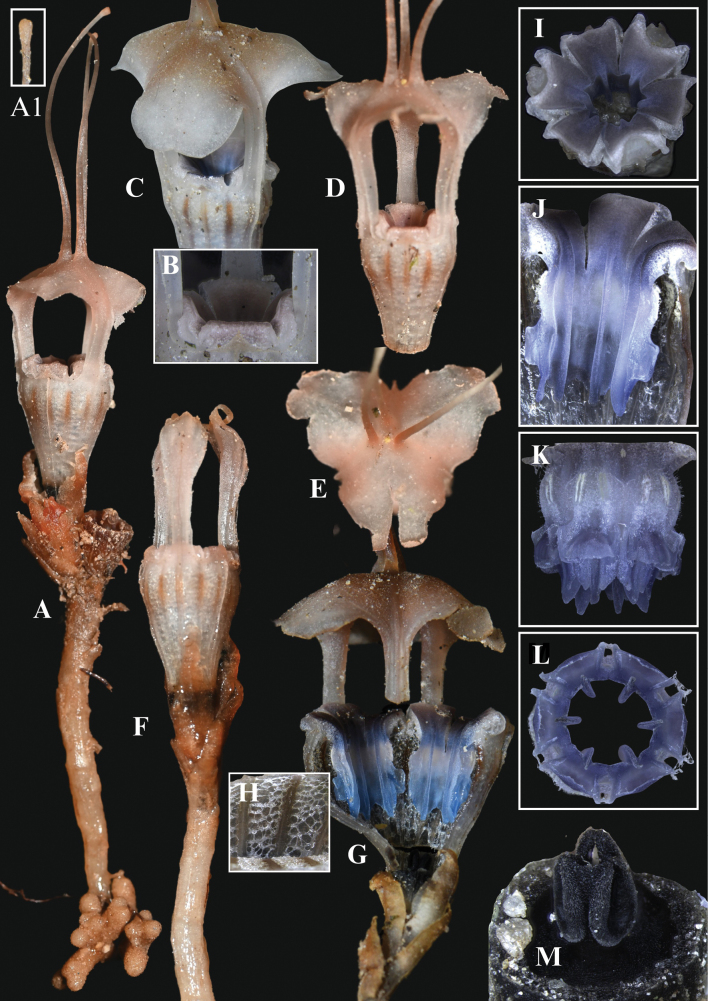
*Thismia selangorensis*. **A.** A plant with full flower and coralliform roots; **A1.** Claviform tip of mitre appendage; **B.** Outer tepal and filaments; **C.** Inner tepal forming a mitre; **D.** Floral tube and mitre; **E.** Mitre viewed from above; **F.** Floral tube with ovary and flower bud surrounded by bracts and tepals split on apex; **G.** Longitudinal section of floral tube, showing inner part (**H**); **H.** Inner surface of floral tube; **I.** Top view of stamens; **J.** Inner view of a stamen; **K.** Outer view of a stamen; **L.** Six stamens viewed from below; **M.** Stigma. Photos by Siti-Munirah MY and Mohd Faizal; all from FRI 79179 & 79180; images not to scale (see dimensions in description and Fig. 2).

**Figure 4.**
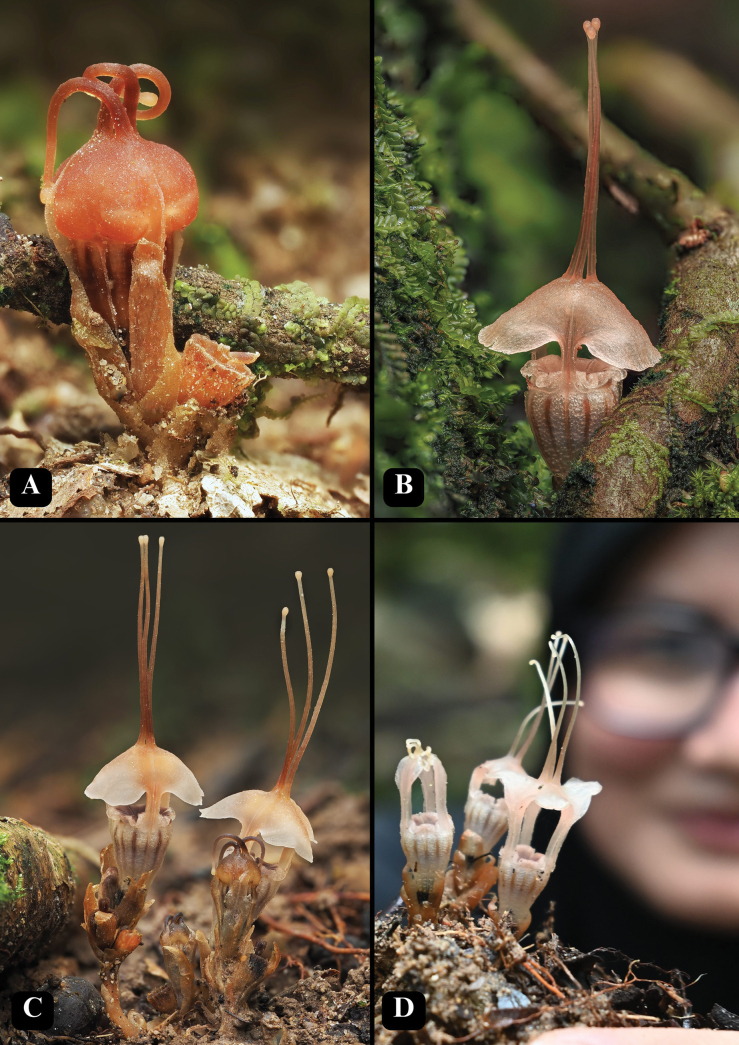
*Thismia selangorensis* in its natural habitat. **A.** Young flower that is not yet fully developed (uncollected plant); **B.** Mature flower living just beside the roots of a tree buttress (uncollected plant); **C.** A clump of *T. selangorensis* at different stages in its natural habitat (FRI 79182); **D.** A clump of flowers showing a different stage of mitre (FRI 79179 & FRI 79180). Photos by Gim Siew (**A–C**) and Mohd Faizal (**D**).

#### Additional specimens examined

**(paratypes).** Peninsular Malaysia: Selangor, Hulu Langat District, TER Sungai Chongkak, ca. 184 m elev., Siti-Munirah MY, FRI 79180 (KEP, spirit collection SC13205), FRI 79182 (KEP, spirit collection SC13206).

#### Distribution.

Endemic to Peninsular Malaysia, Selangor. Currently only known from the type locality.

#### Ecology and habitat.

**Figure 5.**
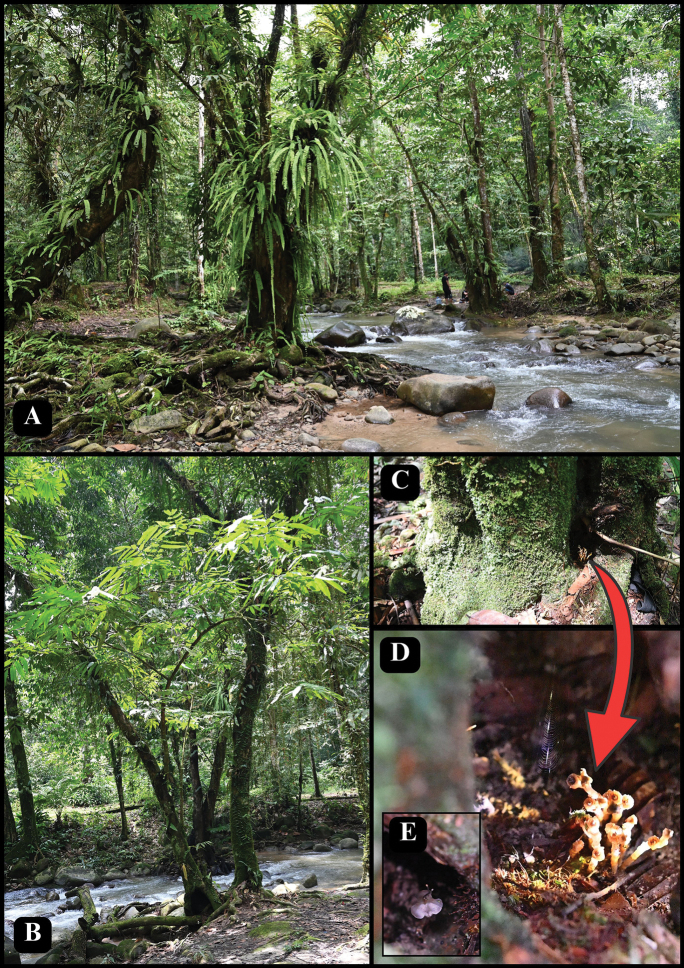
*Thismia selangorensis* habitat at TER Sungai Chongkak. **A.** Main river of TER Sungai Chongkak and a picnic site; **B.***T. selangorensis* type locality area; **C–E.***T. selangorensis* living in trunk base (red arrow) beside the river; **D.** Plant in July 2024; **E.** Plant in November 2023. Photos by Siti-Munirah MY (**A, C–E**) and Mohd Faizal (**B**).

In lowland forests, on moist soil in the closed canopy, hidden between tree buttresses, also in tree hollows, near an open area (picnic site; Fig. 5). This species occurs in a river basin at an altitude of approximately 184 m. The site is located in a public recreational forest managed by the Selangor Tourism Authority and the Forestry Department within a watershed/riverine vegetation. The flowering season is from October to February. The forest area is a major tourist attraction for picnicking and camping. Apart from where the plant was found, part of the area looks like an intact lowland dipterocarp forest. The areas near the river are all flat and have places for a campsite. Fortunately, a few known subpopulations of *T. selangorensis* exist on the riverbank and near tree roots; thus, there is no known direct impact from the campground or nearby playground. However, the status of these populations may also be uncertain because activities occurring along the river could result in trampled plants.

#### Etymology.

The epithet refers to the state, Selangor, where it was found.

#### Conservation status.

*Thismia selangorensis* is a newly described species found in TER Sungai Chongkak, a recreational forest in Selangor. The species was discovered growing in moist, shaded areas near tree buttresses along the riverside. Since its initial discovery in 2023, several surveys have been conducted in the area, and fewer than 20 individual plants were recorded. All the individuals found form a single population sensu IUCN (2024). While the extent of occurrence (EOO) cannot be calculated from a single point, the area of occupancy (AOO) is formally estimated at 4 km^2^. This species appears to be rare and only blooms at certain times of the year. It is mycoheterotrophic and typically only visible when flowering. Its flowers are often small, inconspicuous, and hidden beneath leaf litter or root buttresses. *Thismia selangorensis* is fragile and vulnerable to disturbances due to its small size. It is particularly threatened by trampling, especially in the main picnic area. Plants growing near the river are also at risk of being destroyed by flooding during heavy rain. Consequently, according to the IUCN (2024), this species is assessed as Critically Endangered based on B2ab(iii, v), D.

#### Notes.

In some species of the genus *Thismia*, the inner tepals are fused together in the upper part and form a roof-, hat-, or umbrella-like structure known as a mitre. These species are divided into two groups: 1.) species with mitral appendages (Thismia section Geomitra and also Thismia sect. Scaphiophora); 2.) species without appendages (Thismia section Sarcosiphon). Based on the infrageneric classification of Kumar et al. (2017), *Thismia selangorensis* belongs to Thismia subgenus Thismia, section Geomitra (Becc.) Kumar & S.W. Gale. This classification is primarily based on the presence of inner tepals that form a mitre with three filiform appendages. According to Shepeleva et al. (2020), *T. selangorensis* is possibly related to the species forming Clade 3 due to its coralliform roots, fused inner tepals forming a mitre, and free mitral appendages extending from a central point. Currently, a total of seven species are assigned to section Geomitra (Table 1). In terms of species diversity within *Thismia*, this section represents a small group compared to other sections. To date, all known species of this section are distributed exclusively in Southeast Asia. Of the *Geomitra* species, only five – *T. clavigera*, *T. clavigeroides*, *T. limkokthayi*, *T. kelantanensis* and *T. selangorensis* – are currently found in Malaysia, with the latter three species being endemic to Peninsular Malaysia and known only from their type localities.

**Table 1.** Species of Thismia section Geomitra and their distribution (Chantanaorrapint and Chantanaorrapint 2009; Tsukaya and Okada 2012; Siti-Munirah 2018; Suetsugu et al. 2018; Chantanaorrapint and Seelanan 2021; Siti-Munirah et al. 2022).

No.	Species	Distribution	Endemism
1.	*T. betung-kerihunensis* Tsukaya & H. Okada	Kalimantan (Indonesia)	Endemic
2.	*T. clavigera* (Becc.) F. Muell.	Kedah and Sarawak (also in Thailand)	No
3.	*T. clavigeroides* Chantanaorr. & Seelanan	Terengganu (also in Thailand)	No
4.	*T. selangorensis* Siti-Munirah & Gim Siew	Sungai Chongkak, Selangor	Endemic
5.	*T. limkokthayi* Siti-Munirah & E. Chan	Genting Highlands, Pahang	Endemic
6.	*T. kelantanensis* Siti-Munirah	Gunung Chamah, Kelantan	Endemic
7.	*T. sumatrana* Suetsugu & Tsukaya	Sumatra (Indonesia)	Endemic

Morphologically, the species of section Geomitra can be easily distinguished from those of other *Thismia* sections by the presence of a mitre with three long appendages. In *Geomitra* species, the appendages are generally similar in shape and size. However, the mitre formed by the inner tepal lobes varies and produces different shapes, such as flat, concave, cap-like, umbrella-like, or bonnet-like structures. In Peninsular Malaysian species, flower colouration is also species-specific. Besides that, another important distinguishing feature of all *Geomitra* species compared with other *Thismia* species is the stamen morphology. All *Geomitra* species have a central rib extension in the middle of each stamen. The same stamen morphology, characterised by a central rib extension in the middle of each stamen, is also found in *T. goodii*, *T. kelabitiana*, and *T. coronata* (Dančák et al. 2020b). This means it is not a unique trait of species of Thismia sect. Geomitra, and the *T. goodii* group is probably closely related to T. sect. Geomitra species. However, the supraconnective apex of the stamens differs morphologically among T. sect. Geomitra species, being either strongly different in itself (e.g. *T. limkokthayi*) or only slightly different from each other (e.g. between *T. clavigeroides* and *T. selangorensis*).

In summary, it can be stated that *T. selangorensis* is easily recognised by a combination of the following characteristics: coral-shaped roots; mitriform inner tepals with three erect claviform appendages; absence of annulus; absence of transverse bars on the inner side of the floral tube but presence of reticulation on the inner surface; stamens with a central rib extension (convex on the inner surface, concave on the outer surface); two lateral lobes (each with pointed tips); and a blackish stigma. *Thismia selangorensis* shows different morphological features when compared with closely related species. A detailed comparison can be found in Table 2.

**Table 2.** Morphological differences between *T. selangorensis* and related species (Tsukaya and Okada 2012; Chantanaorrapint and Chantanaorrapint 2009; Siti-Munirah 2018; Suetsugu et al. 2018; Siti-Munirah et al. 2022).

Characters	*T. selangorensis*	*T. limkokthayi*	*T. betung-kerihunensis*	*T. clavigera*	*T. clavigeroides*	*T. kelantanensis*	*T. sumatrana*
**Floral tube colour**	White to brownish	Black-brownish/ brown-blackish	White with indigo and brown to pale brown with purple-dark blue	White-orange	Whitish	Pale to bright and dark blue-purplish translucent	Unknown
**Mitre colour**	White to brownish/peach	Black-brownish/ brown-blackish	Blue-green	Yellowish-orange	Pale brown or grey	Yellow to bright orange	Unknown
**Inner tepal lobe colour**	White to brownish/peach	Brown-blackish	Blue-green	Orange	Pale brown or grey	Bright orange	Unknown
**Outer tepal**	Erect	Erect	Erect	Erect	Reflexed	Erect	Reflexed
**Inner tepal appendages colour**	Brown and pale (dark when young)	Dark brown to pale orange	Pale blue tinged with orange	Orange	Pale brown or grey below, blue-green at the tip	Orange	Unknown
**Mitre fovea**	Absent	Present	Absent	Absent	Absent	Absent	Absent
**Filament colour**	Dark-grey-whitish to bluish	Orange and white	Blue-green	Orange	White	Bright blue	Unknown
**Apex of supraconnective**	One central lobe and two smaller side lobes	One central lobe and two smaller side lobes with recurved tips/truncate, glabrous	One central lobe and two smaller side lobes, hairy	Acute	One central lobe and two smaller side lobes	Acuminate	Acute, hairy
**Presence of transverse bars**	Absent	Absent	Present	Present	Present	Present	Present

### ﻿Key to *Thismia selangorensis* and related species

Modified from Chantanaorrapint and Seelanan 2021.

**Table:** 

1	Supraconnective apex long, distinctly exceeding the lateral appendage, triangular in outline	**2**
–	Supraconnective apex short, of the same length or slightly longer than lateral appendage, trilobed to truncate	**5**
2	Supraconnective apex bearing 1 triangular lobe; stigma lobe entire	**3**
–	Supraconnective apex bearing 3 triangular lobes (mid-lobe larger and two smaller side lobes); stigma lobe bifid	**4**
3	Mitre with 6 hood-like appendages; supraconnective apex long acuminate	***T. kelantanensis***
–	Mitre with 3 hood-like appendages; supraconnective apex acute	***T. sumatrana***
4	Mitre appendages less than 1.5 cm long	***T. clavigera***
–	Mitre appendages more than 3 cm long	***T. selangorensis***
5	Mitre hood present, colour greenish blue	***T. betung-kerihunensis***
–	Mitre flat, colour white to fully blackish	**6**
6	Flower almost black, supraconnective apex recurved inwards, truncate	***T. limkokthayi***
–	Flower mostly white, supraconnective apex straight, trilobed	***T. clavigeroides***

## Supplementary Material

XML Treatment for Thismia selangorensis

## ﻿Additional information

### Use of AI

No use of AI was reported.

### Author ORCIDs

Mat Yunoh Siti-Munirah https://orcid.org/0000-0002-5062-9988

Mohd Faizal Mat-Tahir https://orcid.org/0000-0002-9928-4933

### Data availability

All of the data that support the findings of this study are available in the main text.
